# Gold nano-particles (AuNPs) carrying anti-EBV-miR-BART7-3p inhibit growth of EBV-positive nasopharyngeal carcinoma

**DOI:** 10.18632/oncotarget.3046

**Published:** 2015-02-19

**Authors:** Longmei Cai, Jinbang Li, Xiaona Zhang, Yaoyong Lu, Jianguo Wang, Xiaoming Lyu, Yuxiang Chen, Jinkun Liu, Hongbing Cai, Ying Wang, Xin Li

**Affiliations:** ^1^ Cancer Research Institute, Southern Medical University, Guangzhou 510515, China; ^2^ School of Chinese Traditional Medicine, Southern Medical University, Guangzhou 510515, China; ^3^ Central Medical Laboratory, The Third Affiliated Hospital, Southern Medical University, Guangzhou 510515, China; ^4^ The Sixth Affiliated Hospital of Sun Yat-Sen University, Guangzhou 510655, China; ^5^ Department of Radiation Oncology, Gaozhou People's Hospital, Gaozhou 525200, China

**Keywords:** Nasopharyngeal carcinoma, EBV-miR-BART7, tumorigenesis, therapeutic experiment

## Abstract

Epstein-Barr virus (EBV) infection is a major etiological factor for nasopharyngeal carcinoma (NPC). Several EBV-encoded BART miRNAs have been associated with viral latency, immune escape, cell survival, cell proliferation and apoptosis. Here, we report that EBV-miR-BART7-3p, an EBV-encoded BART miRNA highly expressed in NPC, was correlated with cell-cycle progression *in vitro* and increased tumor formation *in vivo*. This viral miRNA stimulated the PTEN/PI3K/Akt pathway and induced c-Myc and c-Jun. Knockdown of PTEN mimicked EBV-miR-BART7-3p-induced tumorigenic phenotype. Based on these results, we conducted a therapeutic experiment by using gold nano-particles (AuNPs) carrying anti-EBV-miR-BART7-3p. Silencing of EBV-miR-BART7-3p reduced tumor growth in animal model. We conclude that EBV-miR-BART7-3p favors carcinogenesis, representing a potential target for miRNA-based therapy.

## INTRODUCTION

Nasopharyngeal carcinoma (NPC) is a malignancy derived from nasopharyngeal epithelium and is highly prevalent in Southern China and Southeast Asia. Although radiotherapy can effectively control early stage NPC, the average 5-year survival rate after therapy is less than 30–40% in the patients with advanced NPC [1]. Most seriously, due to no obvious symptoms at early stage, more than 60% of clinical NPC patients are easily overlooked and reach advanced stages [2, 3]. Thus, it is necessary to discover novel therapeutic targets and interventions for effectively treating NPC.

MicroRNAs (miRNAs) are endogenous non-coding RNAs that suppress gene expression post-transcriptionally by targeting the 3′UTRs of specific mRNAs. They have emerged as important regulators of physiology and disease, and presented the important therapeutic potential. Recently, aberrant expression of some miRNAs, such as miR-184 [4], miR-34b [5, 6], miR-92a [7], miR-93 [8], miR-27a [9], miR-101 [10], miR-497 [11], and miR-663 [12], has been linked to various cancers including NPC [13], relating to multiple cellular responses such as cell proliferation, cell-cycle progression and tumorigenesis [14, 15]. Epstein-Barr virus (EBV), as a major etiological factor for NPC, encodes 25 miRNA precursors including 3 BHRF1 pre-miRNAs and 22 BART pre-miRNAs [16, 17]. Investigations gradually disclose that this virus can actively deploy its BART miRNAs to flexibly manipulate various viral and host cellular functions. For example, EBV-miR-BART1 influences multiple metabolism–associated genes in NPC [18]. EBV-miR-BART2 suppresses viral DNA polymerase BALF5 to reduce EBV lytic replication [19]. Both EBV-miR-BART5–5p and miR-BART19–5p inhibit viral oncoprotein LMP1 expression [20], while EBV-miR-BART22 decreases LMP2A [21]. Moreover, EBV-miR-BART15–3p suppresses an apoptosis inhibitor BRUCE to induce apoptosis [22] and miR-BART3* targets a tumor suppressor DICE1 to promote cellular growth and transformation in NPC [23]. These findings open up a promising therapeutic option and encourage more studies to develop novel miRNA-based therapies for NPC.

As a member of BART-miRNA family, EBV-miR-BART7-3p is emerging as one of the most important regulatory factors in NPC. We have recently discovered that highly expressed EBV-miR-BART7-3p was closely associated with NPC metastasis and EMT [24]. Other two investigations reported the high EBV-miR-BART7-3p expression in NPC tissue [25]/plasma samples [26] and its correlation with NPC cell proliferation *in vitro* [26], suggesting its involvement in NPC tumorigenesis though the underlying molecular mechanism still remains unclear. In the present study, we not only elucidate the effect of this viral miRNA on NPC tumorigenesis *in vitro* and *in vivo* and explore the underlying regulatory mechanism, but also further investigate the feasibility of using EBV-miR-BART7-3p as a potential onco-target for therapy against NPC. We successfully construct nano-particles carrying anti-EBV-miR-BART7-3p, and carry out an *in vivo* tumor therapy. This may help to understand a viral miRNA-dependent NPC tumorigenesis and provide a promising bio-therapeutic target for this disease.

## RESULTS

### EBV-miR-BART7-3p promoted NPC cell growth and tumorigenesis

**Figure 1:**
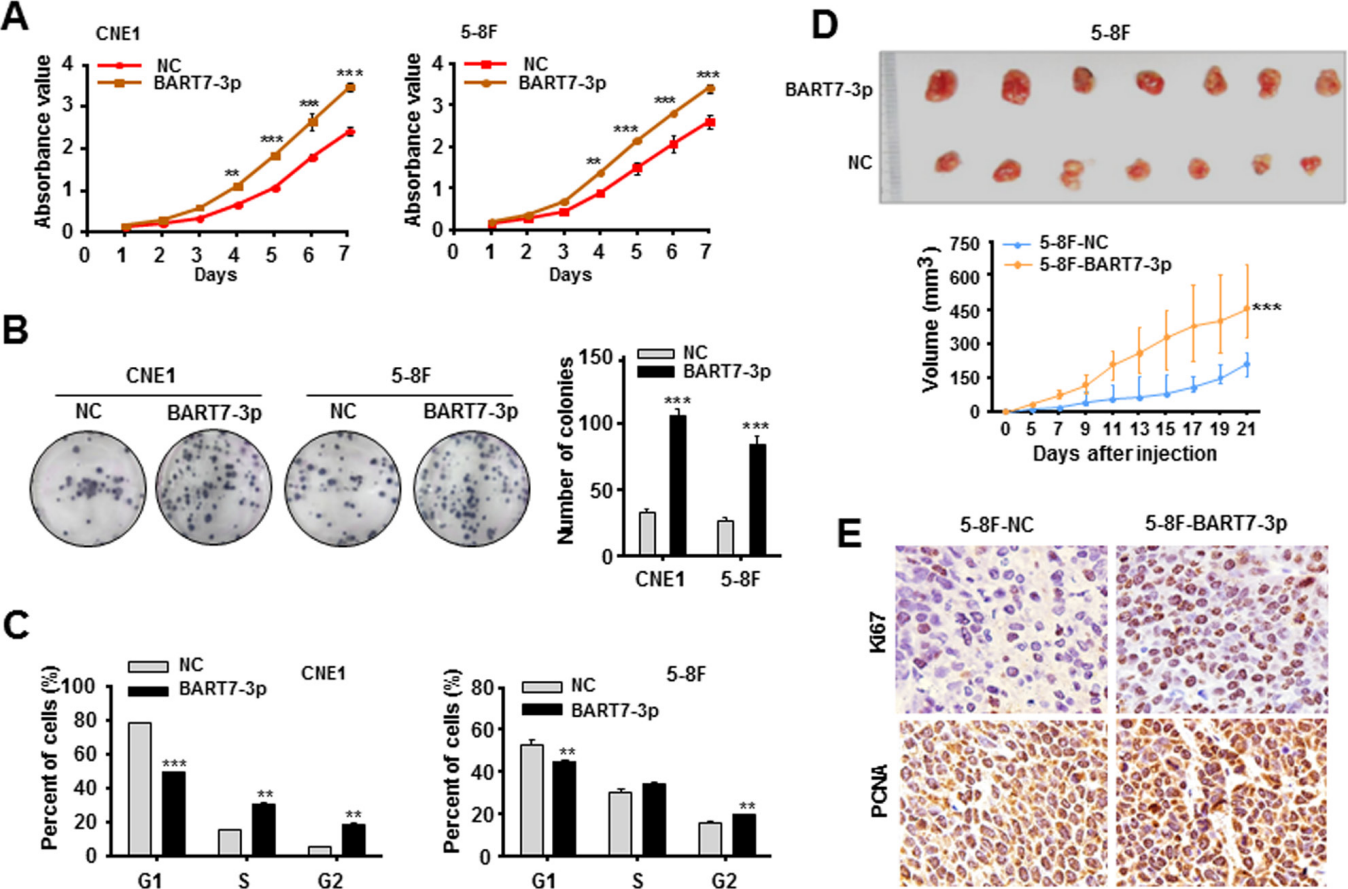
EBV-miR-BART7-3p promotes NPC cell growth and tumorigenesis **(A, B)** MTT assay and colony-formation assay were conducted to evaluate cell growth and proliferation in CNE1 and 5-8F cells stably expressing EBV-miR-BART7-3p (BART7-3p for short) and their correspondence NC cells. The data were shown as the mean ± SEM (**P < 0.01, ***P < 0.001). **(C)** The cell-cycle distribution was detected by the flow cytometry in two stable cell lines as compared with control cells. The data were shown as the mean ± SEM (**P < 0.01, ***P < 0.001). **(D)** *In vivo* tumor formation experiment was conducted by the subcutaneous injection of 5-8F-BART7-3p cells and 5-8F-NC cells into seven nude mice respectively. Top, the nude mouse tumors after 21-day inoculation. Bottom, tumor volumes of each group during the tumor growth, plotted as mean values ± SEM (***P < 0.001). **(E)** The IHC detection of Ki67 and PCNA in the tumor tissues originated from the mouse models.

To gain an insight into the role of EBV-miR-BART7-3p in the cell growth and tumorigenesis of NPC, we firstly generated two NPC cell lines (CNE1 and 5-8F) stably expressing EBV-miR-BART7-3p (5-8F-BART7-3p and CNE1-BART7-3p) and two corresponding control cells (5-8F-NC and CNE1-NC) (Figure S1A, S1B, see materials and methods). EBV-miR-BART7-3p expression levels in these two stable cell lines were in a similar physiological range to NPC tissue samples (Figure S1A, S1B). Subsequently, MTT and colony formation assays showed that this viral miRNA significantly promoted cell growth, proliferation (Figure 1A) and colony formation (Figure 1B). Flow cytometric evaluation displayed that these two stable cell lines had an increased proportion of G2 phase and a decreased proportion of G1 phase relative to the control cell lines (Figure 1C, Figure S2A). Contrarily, after the transfection with EBV-miR-BART7-3p inhibitor (anti-miR), these two stable cell lines presented an obviously reduced cell growth and colony formation (Figure S3A and S3B) as well as an increased G1 phase and fewer cells in G2 phase (Figure S2B and Figure S3C).

Further, we conducted an *in vivo* tumor formation experiment by subcutaneously injecting 5-8F-BART7-3p or 5-8F-NC cells into nude mice. Notably, in three weeks after implantation, the mice injected with 5-8F-BART7-3p cells appeared to carry larger tumor burdens (Figure 1D, Figure S4) and display relatively higher expression levels of Ki67 and PCNA in tumor tissues relative to the controls (Figure 1E).

We also examined EBV-miR-BART7-3p expression in 40 clinical NPC specimens. EBV-miR-BART7-3p tended to be highly expressed in NPC patients with advanced T classification (T3-T4) (Figure S5A), hinting it may be a late event involving in NPC tumorigenesis.

Collectively, these results indicated that EBV-miR-BART7-3p had the ability to enhance NPC tumorigenesis.

### EBV-miR-BART7-3p enhanced cell growth and proliferation via stimulating the PTEN/PI3K/Akt pathway and inducing c-Myc and c-Jun

We previously found that EBV-miR-BART7-3p promoted NPC metastasis and EMT via suppressing Phosphatase and tensin homolog (PTEN) [24]. In this present study, we further discovered a new binding site for this miRNA in the 3′UTR of PTEN (Figure S6A). Luciferase reporter assay showed that EBV-miR-BART7-3p mimic attenuated the fluorescence intensity of reporter vector with wt 3′UTR of PTEN by more than two-fold relative to NC, whereas mut 3′UTR showed no response to mimic. The reduced fluorescence intensity of reporter vector with wt 3′UTR was rescued by anti-miR (Figure S6B). Consistently, upon immunohistochemistry evaluation of PTEN protein expression in tissue samples derived from *in vivo* tumor formation models, we also found an obvious reduction of PTEN expression in 5-8F-BART7-3p group relative to control (Figure S6C). Theses data provided additional evidence that this miRNA repressed PTEN by directly binding to its 3′UTR (Figure S5B).

**Figure 2:**
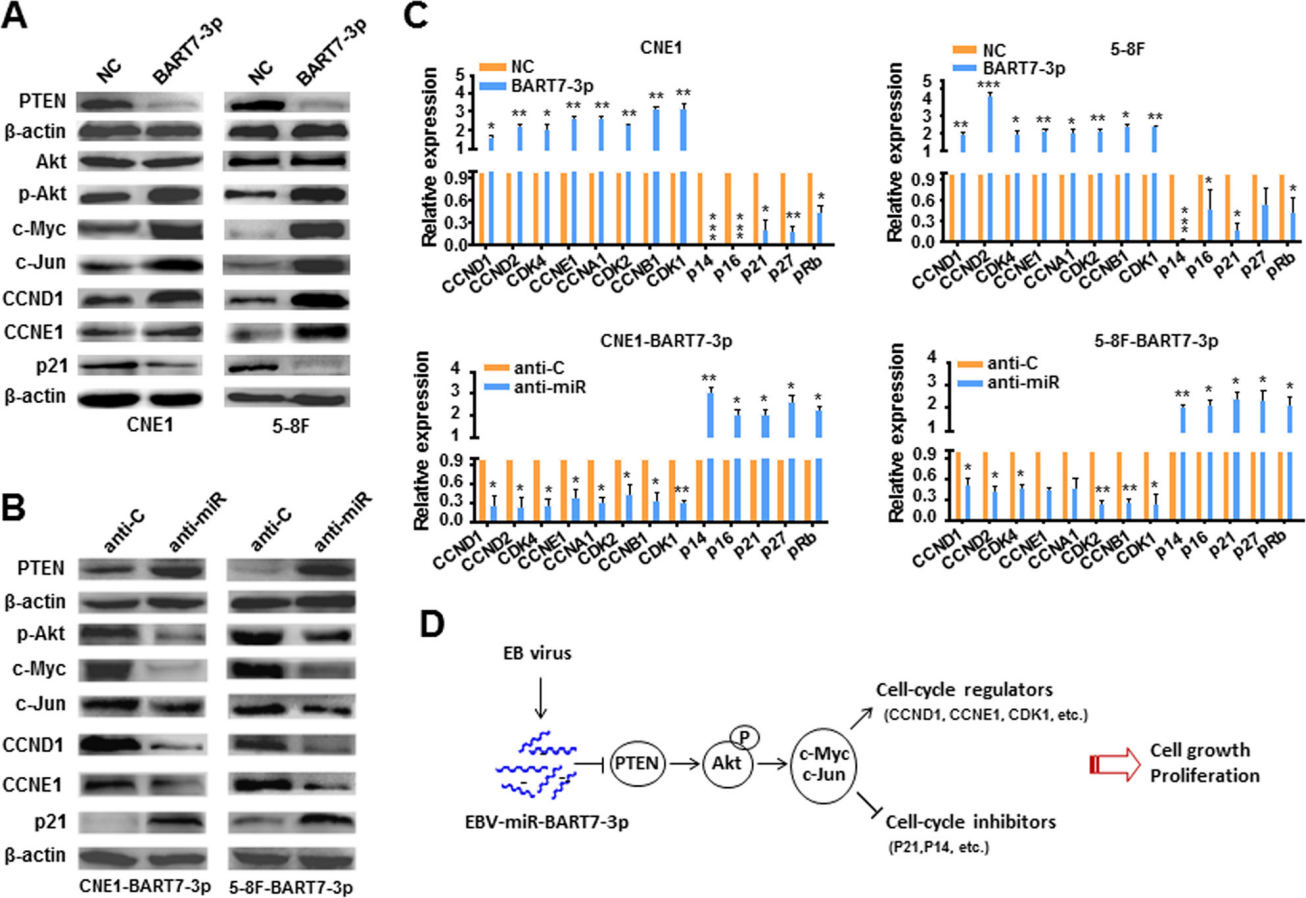
EBV-miR-BART7-3p stimulated PTEN/PI3K/Akt pathway and its downstream signals **(A)** The expression levels of Akt, p-Akt, c-Myc, c-Jun, CCND1, CCNE1 and p21 were detected using western blotting in 5-8F-BART7-3p and CNE1-BART7-3p cells or **(B)** after transfected with anti-miR or anti-C. β-actin served as an internal control. **(C)** The mRNA expression levels of cell-cycle regulators were analyzed by qPCR in 5-8F-BART7-3p and CNE1-BART7-3p cells before and after transfected with anti-miR. All data were normalized to GAPDH expression and plotted as mean values ± SEM (*p < 0.05, **p < 0.01, ***p < 0.001). **(D)** The diagram of EBV-miR-BART7-3-mediated signaling pathway for the cell growth and proliferation in NPC.

PTEN/PI3K/Akt constitutes an important pathway modulating multiple biological processes. To address the mechanism of EBV-miR-BART7-3p-mediated phenotypic changes, we conducted western blotting assay to examine phosphorylated Akt (Ser473), a centrally important effector of this pathway. We observed that EBV-miR-BART7-3p enhanced p-Akt expression via suppressing PTEN expression, whereas anti-miR-BART7-3p rescued its expression (Figure 2A and 2B). More interestingly, the expression levels of c-Myc and c-Jun, two transcriptional factors that favor cell growth and proliferation of cancers [27–29], were also increased accordingly (Figure 2A and 2B), indicating that EBV-miR-BART7-3p stimulated PI3K/Akt/c-myc and c-Jun through suppressing PTEN in NPC cells (Figure 2A and 2B). Following the observation of EBV-miR-BART7-3p-mediated growth promotion, we next evaluated the expression of cell-cycle associated genes in EBV-miR-BART7-3p overexpressing or suppressing cells. Two cell-cycle regulators (CCND1 and CCNE1) were highly expressed in EBV-miR-BART7-3p overexpressing cells and lowly expressed in EBV-miR-BART7-3p suppressing cells, whereas P21^CIP1^, a cell-cycle inhibitor, was lowly expressed in EBV-miR-BART7-3p overexpressing cells and highly expressed in EBV-miR-BART7-3p suppressing cells (Figure 2A and 2B), suggesting the effects of EBV-miR-BART7-3p on cell cycle process probably through activating Akt/c-myc and c-Jun. Furthermore, we did an expanded qPCR examination of cell-cycle regulators and inhibitors. The levels of cyclins and cyclin-dependent kinases were generally increased more than 2-fold upon EBV-miR-BART7-3p overexpression, whereas the levels of cell cycle inhibitors were decreased at least 2-fold (Figure 2C). The opposite results appeared in 5-8F-BART7-3p and CNE1-BART7-3p cells after treating with anti-BART7-3p (Figure 2C). Therefore, these data indicated that EBV-miR-BART7-3p stimulated the PTEN/PI3K/Akt signaling pathway and induced c-Myc and c-Jun, thereby influencing cell cycle process and eventually promoting cell growth and proliferation of NPC (Figure 2D).

**Figure 3:**
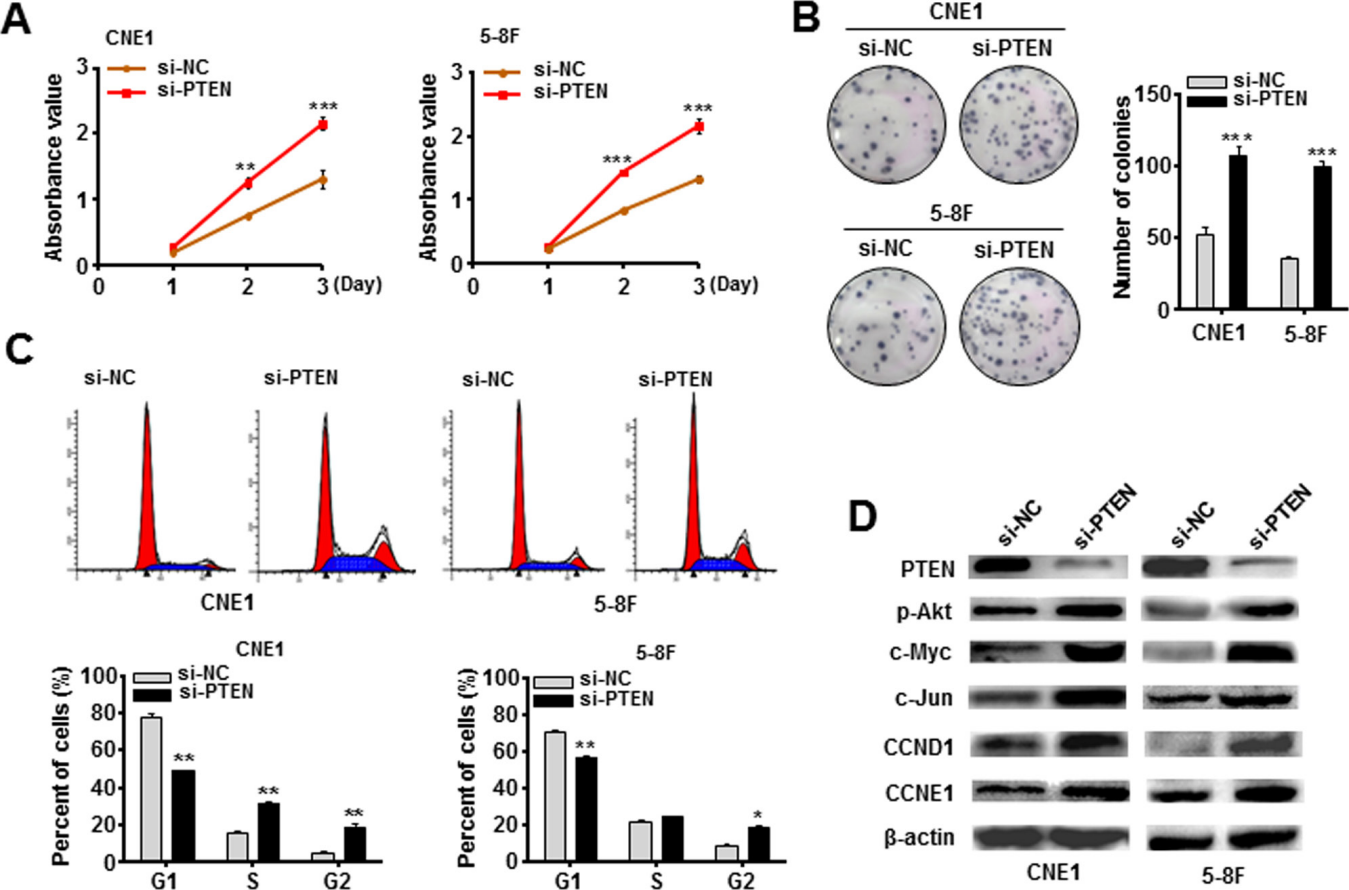
Knockdown of PTEN mimicked the EBV-miR-BART7-3p-induced phenotype in NPC cells **(A, B)** Cell growth and proliferation ability was detected by MTT assay and in colony formation assay in CNE1 and 5-8F cells following the treatment of siRNA against PTEN (si-PTEN for short) or si-NC (Control). Data were shown as the mean ± SEM (**P < 0.01, ***P < 0.001). **(C)** The cell-cycle transition from G1 to S and G2 was evaluated by the flow cytometry after PTEN was silenced *in vitro*. Data were shown as the mean ± SEM (*P < 0.05, **P < 0.01). **(D)** The expression levels of PTEN, p-Akt, c-Myc, CCND1 and CCNE1 were analyzed by western blot in indicated cells after treated with si-PTEN. β-actin is internal control.

The siRNA against PTEN (si-PTEN) was also transfected into CNE1 and 5-8F cells. As expected, si-PTEN-transfected CNE1 and 5-8F-cells presented a higher ability to grow and proliferate relative to the control cells (Figure 3A and 3B). Similarly, flow cytometry analysis revealed an increased percentage of G2 phase cells in two NPC cell lines after introducing si-PTEN (Figure 3C). Western blotting assay confirmed a reduced PTEN protein expression in both CNE1 and 5-8F cells after 72 h transfection of si-PTEN (Figure 3D), followed by an obviously induced expression of p-Akt, c-Myc, c-Jun, CCND1 and CCNE1 (Figure 3D). These results supported that knockdown of PTEN mimicked the EBV-miR-BART7-3p-induced tumorigenic phenotype.

### Silencing of EBV-miR-BART7-3p reduced the *in vitro* growth of EBV-positive NPC cells

**Figure 4:**
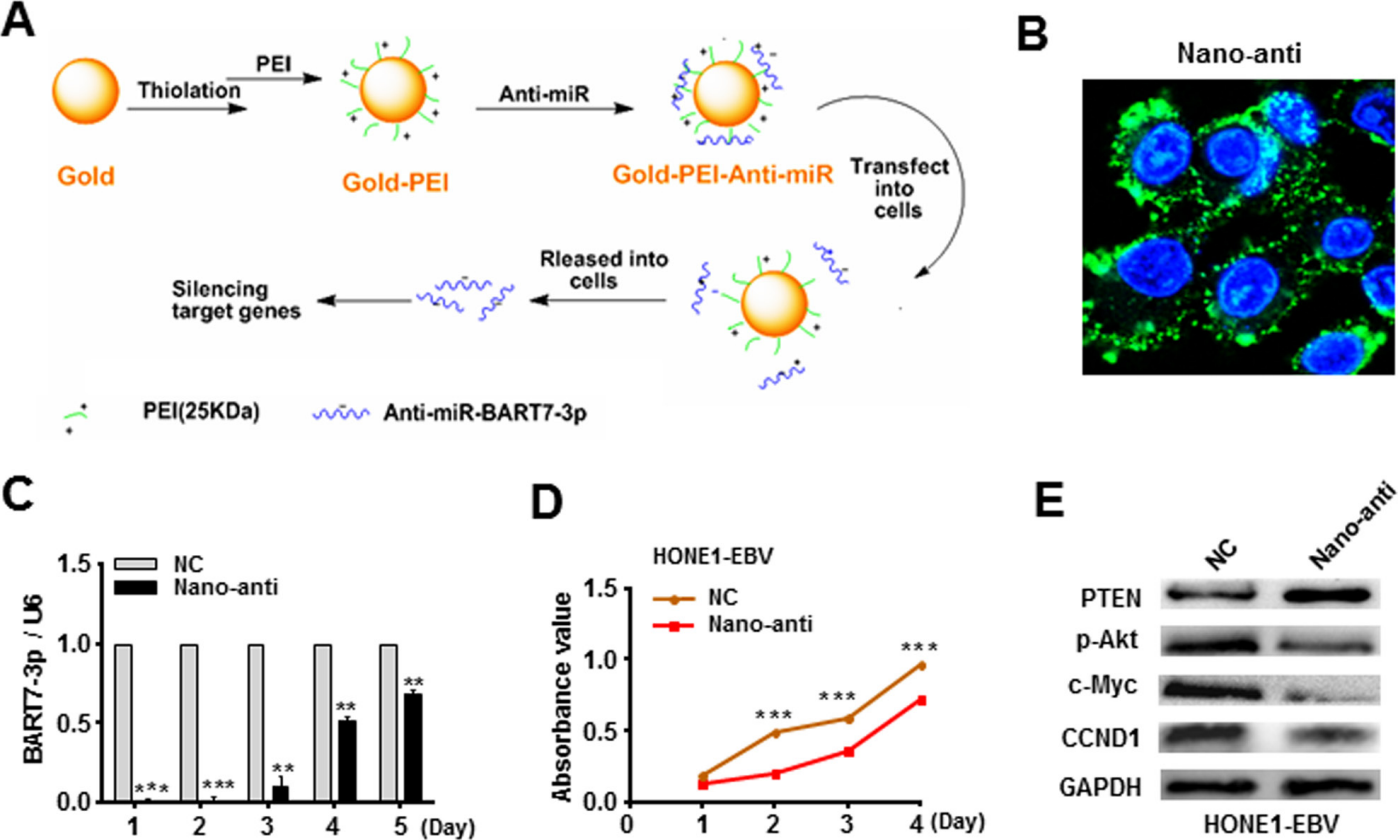
Silencing of EBV-miR-BART7-3p reduced the *in vitro* growth of EBV-positive NPC cells **(A)** A layer-by-layer method was applied to prepare gold-PEI nanoparticles carrying anti-miRNA. **(B)** The transfection efficiency of nanoparticles carrying anti-miRNA was evaluated by confocal microscopy at cellular level. **(C)** The inhibition efficiency of nano-anti-miR in HONE1-EBV cells was evaluated by qPCR in different time points. Data were shown as the mean ± SEM (**P < 0.01, ***P < 0.001). **(D)** Cell growth ability was evaluated by MTT assay in HONE1-EBV cells after transfected with anti-miR or anti-C. Data were shown as the mean ± SEM (***P < 0.001). **(E)** The expression levels of PTEN, p-Akt, c-Myc and CCND1 were analyzed by western blot in HONE1/EBV cells after transfected with anti-miR or anti-C. β-actin served as an internal control.

Given that EBV-miR-BART7-3p modulated growth promotion, we further explored whether or not EBV-miR-BART7-3p was a potential therapeutic target for NPC. We firstly analyzed EBV-miR-BART7-3p expression by qPCR in several EBV-positive NPC cell lines. As shown in Figure S7, there was a similar expression level of EBV-miR-BART7-3p in EBV positive NPC cells to the pooled NPC tissues. Of these EBV-positive NPC cell lines, HONE1-EBV cell line was selected as a representative cellular model because it grew better and was suitable for the following *in vitro* and *in vivo* experiments [24]. Subsequently, we fabricated a nano-carrier (gold-PEI) using a layer-by-layer method [30] (Figure 4A, see materials and methods) to deliver anti-EBV-miR-BART7-3p into HONE1-EBV cells. The size of Gold-PEI nano-carrier was about 20–30 nm as measured by dynamic lighting scatter (DLS) (Figure S8A). The zeta potential of gold-PEI was positive charge (about 20 mV) that facilitated the absorption of anti-miR with negative charge (Figure S8B). The nano-anti-miR displayed an approximately spherical shape with good dispersion and its size was similar to that of gold-PEI observed by TEM (Figure S9). To validate the transfection efficiency at the cellular level, we next investigated the cellular uptake of nano-carrier with FAM-anti-miR using confocal microscopy. There were much more visible green fluorescence particles in HONE-EBV cells transfected with nano-anti-miR (Figure 4B). Furthermore, the inhibition efficiency of nano-anti-miR was evaluated. qPCR revealed that nano-anti-miR effectively inhibited EBV-miR-BART-3p expression level in HONE-EBV cells in the first three days (Figure 4C). After treating with nano-anti-miR, cell growth was accordingly reduced (Figure 4D) and PTEN expression was obviously increased in HONE-EBV while the relevant signals (p-Akt, c-Myc and CCND1) were decreased (Figure 4E).

### Silencing of EBV-miR-BART7-3p therapeutically inhibited the tumorigenicity of EBV-positive NPC cells *in vivo*

**Figure 5:**
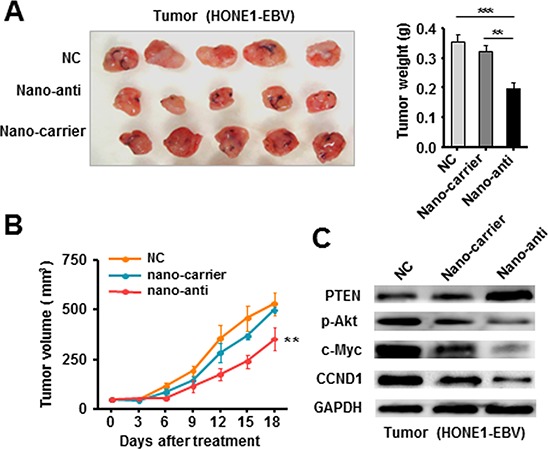
Therapeutically Silencing of EBV-miR-BART7-3p inhibited tumorigenicity of EBV-positive NPC cells *in vivo* **(A)** The *in vivo* effectiveness of nano-anti-miR was evaluated in xenograft mouse models bearing tumors originating from HONE1-EBV cells. **(B)** The tumor volume was periodically tested for each mouse and tumor growth curve was plotted. Data were shown as the mean ± SEM (**P < 0.01, ***P < 0.001). **(C)** The expression of PTEN, p-Akt, c-Myc and CCND1 was analyzed by western blot in the tumor tissues derived from therapeutic and control models respectively. β-actin served as an internal control.

We further evaluated the *in vivo* effectiveness of nano-anti-miR in mice bearing tumors originating from HONE1-EBV cells. The nano-anti-miR was injected into the solid tumor of each mouse per three days according to the results of *in vitro* experiments. During the 18-day treatment, tumor volume was periodically tested and growth curve was plotted. Consistently, we observed that nano-anti-miR obviously inhibited tumor growth compared with NC control or nano-carrier groups (Figure 5A and 5B). Western blot assay confirmed that PTEN expression was also decreased in tumor tissues, whereas p-Akt, c-Myc and CCND1 were increased after the treatment of nano-anti (Figure 5C).

## DISCUSSION

The incidence of viral infection-associated human cancer is rising. Approximately 90% of the people in the world carry latent viruses that can cause various cancers. These viruses majorly include human immunodeficiency virus (HIV), cytomegalovirus (CMV), papilloma virus (HPV), hepatitis B virus (HBV) and EBV [31]. It is estimated that EBV-associated cancers account for about 1.5% of all cancers worldwide, causing about 200,000 cancers worldwide each year, most commonly Burkitt's lymphoma, NPC and about 10% of gastric cancer [32]. Recently, exploring the specific vaccine and therapeutic genes has been becoming a major approach against virus infection [33]. Some tumor-causing viruses such as HPV and HBV have been the subjects of successful vaccination development programs, but there are still no specific treatments or therapeutic genes available for preventing against EBV infection and its associated diseases [31, 34, 35].

EBV is closely associated with NPC. Several EBV-encoded proteins, such as LMP1 [36], LMP2A [37], and BALF3 [38], have been reported to promote the growth and proliferation of NPC cells. However, not all tumor tissues express these viral oncoproteins, suggesting that other viral factors may contribute to NPC aggressiveness. Among the various regulatory mechanisms underlying NPC [39], EBV-encoded miRNAs have been increasingly brought to attention in light of their potential regulatory roles in many aspects [40], including latent virus maintenance [41], escape immune recognition [23], anti-apoptosis [42], and the promotion of invasion and metastasis [43], but there are rare studies on EBV-encoded miRNAs relevant to tumor growth and tumorigenesis. In the present study, the role of EBV-miR-BART7-3p in NPC tumorigenesis was investigated. *In vitro* and *in vivo* experiments revealed that this viral miRNA enhanced cell growth, colony formation and cell-cycle progression of NPC cells, provoking further tumorigenesis. Interferential experiments displayed that its functions were reversed upon using specific anti-miR. Clinical sample analysis showed a positive correlation between EBV-miR-BART7-3p expression and NPC T classification in clinical specimens, consistent with our previous observation that this viral miRNA was positively correlated with lymph node metastasis and clinical stage of NPC [24], further highlighting a clinical effect of EBV-miR-BART7-3p on NPC tumorigenesis. These results demonstrate that EBV-miR-BART7-3p may play roles as an oncomir in the development and progression of NPC.

To date only a few cellular target genes, such as DICE1 [23], CXCL11 [44], PUMA [42] and E-cadherin [43], have been identified for EBV-encoded miRNAs. As a major tumor suppressor, PTEN has been closely associated with cell proliferation [45], differentiation [46] apoptosis [47], and metastasis [48] in various cancers. We previously demonstrated that PTEN was targeted by EBV-miR-BART7-3p in NPC metastasis and EMT [24]. In the present study, we discovered and experimentally confirmed an additional binding site for this viral miRNA within the 3′UTR of PTEN, and observed that silencing of PTEN mimicked EBV-miR-BART7-3p-induced tumorigenic phenotype, providing additional evidence that EBV-miR-BART7-3p did directly inhibit PTEN in NPC tumorigenesis. It is long believed that PTEN and PI3K/Akt constitute an important pathway modulating multiple biological processes. PTEN is a dual protein/lipid phosphatase which major substrate is PIP3, the product of PI3K. PIP3 can recruit Akt to the membrane where phosphorylation by other kinases (such as PDK1) can activate Akt [49, 50]. Particularly, the activation of PI3K/Akt pathway is always responsible for lowly expressed PTEN in cancers including NPC [51, 52]. Here, our experiments also showed a decrease in PTEN expression and an increase in p-Akt expression in the presence of EBV-miR-BART7-3p, indicating that this pathway was stimulated in EBV-miR-BART7-3p-mediated NPC tumorigensis. Interestingly, it is known that c-Myc and c-Jun transcription factors are critical promoters of cellular proliferation. It is in the active Akt-dependent manner that they can be induced though the regulation is probably indirect [49, 53]. These two transcriptional factors can modulate cell-cycle progression [54, 55], potentiating the transition from the G1 phase into the S phase (G1/S transition) with progression through the cell cycle and further continuing cell proliferation [56]. Of note, in the present study, we observed that c-Myc and c-Jun were induced in the presence of EBV-miR-BART7-3p, and several cell-cycle regulators and inhibitors were influenced accordingly. These data first disclose that a viral miRNA may affect cell-cycle progression and promote cell proliferation through activating PI3K/Akt/c-myc and c-Jun.

Disease-associated miRNAs have recently become potential targets for therapeutic intervention [57–59]. An inspiring fact is that delivery of a locked nucleic acid (LNA)-modified oligonucliotide complementary to miR-122 has been successfully applied to decrease HCV viremia in primates [60]. This provides evidence of the feasibility of delivering anti-miRNA oligonucleotides for therapy. Nanotechnology has provided a good platform for cancer gene therapy based on nanoparticle unique properties, for example, diverse surface chemistry, appropriate size scale and special pharmacokinetics [61]. The fabrication of nanosized carrier for the delivery of nucleic acid has gained intensive interest because of their potentially clinical application [62]. In the present study, we verified the tumorigenic function of EBV-miR-BART7-3p in NPC, suggesting it was a potential therapeutic target for NPC treatment. Accordingly, we developed a gold-PEI nanocarrier with good transfection efficiency to deliver anti-miR-BART-3p and elicit its potential therapeutic effect. *In vitro* and *In vivo* data revealed that cell proliferation and tumor growth was effectively suppressed by anti-EBV-miR-BART7-3p transported by nano-particles and the expression of relevant genes was modulated accordingly, indicating the feasibility of utilizing nano-particles to deliver anti-miR and silence endogenous EBV-miR-BART-3p. More importantly, these results had a clinical implication for cancer treatment involving miRNA intervention by nano-delivery, although there may be several limitations. For example, the intratumoral injection may not fully reflect the curative effect of systemic or intranasal administration in mice. Chemically synthesized nanoparticles may have a certain degree of toxicity and instability that may not be fully evaluated in such a therapeutic experiment done using intratumoral injection. Therefore, our group has been developing a spray that can deliver nano-anti-miR directly into nasal cavity, and is planning to generate the plant-derived nano carrying anti-miR. This may facilitate designing a better *in vivo* experiment and even clinical application. Moreover, given that multiple EBV BART miRNAs are highly expressed in NPC, a promising nanoparticle-based system carrying multiple antisenses against EBV BART miRNAs is deserved to be established for NPC treatment in the near future.

In summary, we demonstrated that EBV-miR-BART7-3p was a viral oncomir, highly overexpressing in NPC tissues and promoting NPC cell growth, proliferation and tumorigenesis. It stimulated the PTEN/PI3K/Akt pathway and induced c-Myc and c-Jun. We also developed a gold-PEI nanocarrier to deliver anti-miR-BART-3p and elicit its potential therapeutic effect in animal models. Although miRNA-based therapeutics is still in their infancy, our findings are encouraging and EBV BART miRNAs may be potential therapeutic targets for the treatment of patients with NPC.

## MATERIALS AND METHODS

### Tissue specimens and cell line

A total of 40 NPC specimens (not pretreated with radiotherapy or chemotherapy) with TNM classification and 15 NP specimens were collected and confirmed pathologically in Zhongshan People's Hospital, Guangdong, China for qPCR and clinical data analyses (Table S1 and S2). Staging was performed according to the 1992 Fuzhou NPC staging system of China [63]. T classification (The “T” plus a letter or number 0 to 4) is applied to characterize the size and location of the tumor. Advanced T stage is the late stage (stage T3–4) of NPC relative to the earlier stage (T1 or T2). Clinical tissue studies for research purposes have received patient's informed consents and the approval from the Ethics Committee of Southern Medical University, Guangzhou and Zhongshan People's Hospital, Guangdong, China.

Two EBV-negative NPC cell lines (CNE1, 5-8F) and human 293T cells were obtained from Cancer Research Institute of Southern Medical University, Guangzhou, China. Three EBV-positive NPC cell lines (C666–1-EBV, HONE1-EBV, and HK1-EBV) were kindly offered by Prof. George S. W. Tsao from the University of Hong Kong. All cell lines were cultured in RPMI-1640 (HyClone) with 10% calf serum (Gibco) at 37°C and 5% CO_2_.

### RNA extraction and qPCR

Total RNA was extracted from tissues and cell lines with TRIzol reagent (Invitrogen) according to the user manual. cDNA was synthesized with the PrimeScript RT reagent Kit (TaKaRa). Quantitative PCR analyses were performed with SYBR Premix Ex Taq (TaKaRa) on Mx3005P Stratagene. The primers used are shown in Table S4 and S5. All data were normalized to GAPDH expression and further normalized to negative control unless otherwise indicated. Quantification of EBV-miR-BART7-3p was performed with TaqMan miRNA assays (Applied Biosystems). Mature miRNAs were reverse-transcribed, and real-time PCR was performed using All-in-One™ miRNA qRT-PCR Detection Kit following the manufacturer's protocol (GeneCopoeia™). Data were normalized to small nuclear RNA RNU6B (U6 snRNA) expression. qRT-PCR was conducted for each sample in triplicate. The fold change was calculated through relative quantification (2^−ΔΔCt^).

### Lentivirus production and infection

Lentiviral (GV209, H1-MCS-CMV-EGFP) particles carrying EBV-miR-BART7-3p precursor (BART7-3p for short) and its franking control sequence (NC for short) were constructed by GeneChem, Shanghai, China. The Lentiviral transduction of NPC cells was carried out according to the manufactures' protocol. CNE1 and 5-8F cells were infected with recombinant Lentiviral transducing units plus 8 mg/ml Polybrene (Sigma-Aldrich) for 2 days, then the EBV-miR-BART7-stably-expressed NPC cells, their corresponding NC cells (GFP^+^) were sorted with BD FACS Aria^TM^ cell sorter for the following experiments. The expression of EBV-miR-BART7-3p was validated by qPCR (Figure S1A, S1B).

### RNA oligoribonucleotides and cell transfection

EBV-miR-BART7-3p mimic and inhibitors (anti-miR) (2′-O-methyl modification) were synthesized by Genepharma (Shanghai, China) (Table S3). PTEN siRNA (h2) (Santa Cruz, sc-44272) and its Control siRNA-A (Santa Cruz, sc-37007) were indicated as si-PTEN and si-NC respectively.

Before transfection, the medium was changed to the RPMI-1640 (HyClone) with 10% fetal bovine serum (Gibco). All cells were maintained in a humidified atmosphere of 95% air and 5% CO_2_ at 37°C, and seeded on six-well plates (NEST, China) 24 h prior to transfection. Si-PTEN, EBV-miR-BART7-3p mimic or anti-miR was transfected into cells respectively at a final concentration of 50 nmol/L using Lipofectamine^TM^ 2000 (Invitrogen, 11668–019) in serum-free conditions. Six hours later, the medium was changed to fresh RPMI-1640 (HyClone) with 10% fetal bovine serum (Gibco).

### Cell proliferation analysis

Cell proliferation was analyzed using MTT assay. Briefly, 1 × 10^3^ cells were seeded into a 96-well plate with six replicates for each condition and incubated at 37°C. The cells, stably overexpressing EBV-miR-BART7-3p, were incubated for 1, 2, 3, 4, 5, 6 and 7 days. The cells, infected with anti-miR or si-PTEN, were incubated for 24, 48, and 72 h. 20 ml of MTT (5 mg/ml) (Sigma) was added to each well and incubated for 4 h. At the end of incubation, the media was replaced with 150 ml of dimethyl sulfoxide (DMSO; Sigma). The absorbance value (OD) of each well was measured at 490 nm using a microplate reader. Experiments were performed in triplicate.

### Colony formation assay

Cells were plated in six-well culture plates at 2 × 10^2^ cells/well, and the medium was replaced every 3 days. Each cell group had three wells. After incubation for 12 days at 37°C, cells were washed twice with PBS, fixed with 4% paraformaldehyde, and stained with 0.5% crystal violet. The number of colony formation was counted under a microscope.

### Cell cycle analysis

For cell cycle analysis, 1 × 10^5^ cells were seeded on 6-well plates in RPMI 1640 containing 10% FBS. After incubation for 48 h, a total of 1 × 10^6^ cells were harvested, rinsed with cold PBS, and fixed with 70% ice-cold ethanol at 4°C overnight. Fixed cells were rinsed with cold PBS followed by incubation with PBS containing 10 mg/ml propidium iodide and 0.5 mg/ml RNase for 30 min at 37°C. The DNA content of labeled cells was acquired using FACS cytometry assay (BD Biosciences). Each experiment was performed in triplicate.

### *In vivo* tumorigenesis in nude mice

Animal experiments were approved by the Ethical Committee of Animal Research at Southern Medical University. The experimental protocol was established according to the associated national guidelines from Ministry of Science and Technology of China.

*In vivo* tumorigenic ability of EBV-miR-BART7-3p was investigated by tumor xenograft experiment. A total of 1 × 10^6^ 5-8F-BART7-3p and 5-8F-NC in 0.2 ml RPMI 1640 medium were subcutaneously injected into the dorsal flanks of 4–6-week-old male BALB/c nu/nu mice. The mice were maintained in a barrier facility on HEPA-filtered racks and fed with an autoclaved laboratory rodent diet. Each experimental group contained seven mice. Tumor size was monitored using a calliper in the process of tumor growth and measured every 3 days. All animal studies were conducted in accordance with the principles and procedures outlined in Southern Medical University Guide for the Care and Use of Animals under assurance number SCXK (Guangdong) 2011-020. After 3 weeks, mice were killed and tumors were dissected. Tumor volumes were calculated as follows: volume = (D × d ^2^)/2, where D is the longest diameter and d is the shortest diameter.

### Dual luciferase assay

293T cells (1×10^4^) were cultured in 24-well plates and co-transfected with 20 nM EBV-miR-BART7-3p mimics or NC, 5 ng of pRL-CMV Renilla luciferase reporter and 30ng of luciferase reporter that contained the wild-type or mutant 3′-UTR of PTEN. For antagonism experiments, cells were also co-transfected with 20 nM anti-miR-BART7-3p or anti-C. Transfections were performed in duplicate and repeated in three independent experiments. 48 h after transfection, the luciferase activities were analyzed with a Dual-Luciferase Reporter Assay System (Promega).

### Western blot analysis

Western blotting analyses were performed with standard methods. Briefly, cell pallets were lysed in the radio-immunoprecipitation assay (RIPA) buffer containing protease inhibitors (Sigma-Aldrich) and phosphatase inhibitors (Keygen, China). Proteins were separated by 10% SDS-PAGE gels, and blotted onto PVDF (polyvinylidene difluoride) membrane (Millipore). The membrane was probed with the specific antibodies (Table S6), and then with peroxidase-conjugated secondary antibodies. Beta-actin was used as a protein loading control. The bands were visualized by eECL Western Blot Kit (CWBIO Technology). The images were captured with ChemiDocTM CRS+ Molecular Imager (Bio-Rad).

### Immunohistochemical staining

The paraffin sections prepared from *in vivo* experiments were applied to immunohistochemistry assays for detecting protein expression levels of PTEN, Ki67 and PCNA proteins. The indirect streptavidin-peroxidase method was used as the manufacture's introduction. The immunohistochemically stained tissue sections were reviewed separately by two pathologists. The information of antibodies was shown in table S6.

### Preparation of AuNPs-PEI/PEG-antisense (nano-anti)

AuNPs-PEI/PEG-antisense was prepared as previously described method [30]. Briefly, AuNPs with the average diameter of 20 nm were prepared by using the standard reduction of tetrachloroauric (III) acid with sodium citrate. The pH of AuNPs was adjusted to 10, followed by the addition of 11-MUA at a final concentration of 0.1 mg/mL. The stabilized particles were purified twice at 12,000 xg for 25 min and resuspended in 1 mM NaCl. For coating step with PEI/PEG, 1.0 mg/mL PEI/PEG was added to the AuNPs-MUA, and stirred for 30 min. Purification of crude product was performed twice at 12,000 xg for 25 min and the stabilized particles were resuspended in 10 mM NaCl. For coating step with antisense, antisense was added at a final concentration of 2.0 μM to purified PEI/PEG-AuNPs and reacted for 30 min at room temperature.

### Treatment experiment on nude mice

*In vivo* experiment was approved by the Animal Care and Use Committee of Southern Medical University. All mice of 4–5 weeks old and 16–20 g in weight were provided by the Central Animal Facility of Southern Medical University. To establish an NPC mouse model, 6 × 10^5^ HONE1-EBV cells (expressing EBV-miR-BART7-3p) in 0.2 mL buffered saline were subcutaneously injected into the back of nude mice (BALB/C, nu/nu, 4–6 weeks) and tumors were allowed to grow for 10 days. The tumor volume was measured with a caliper every three days and calculated by the formula: Volume = 1/2 × length × width^2^, where length represented the longest tumor diameter and width represented the shortest tumor diameter. When tumor volume reached 30–100 mm^3^, the animals were randomized into three groups for therapy testing. Mice were intratumorally injected with saline, nano-carrier (10 uL) and nano-anti (10 μL nano-carrier combined with 8 μL EBV-miR-BART7-3p antisense), respectively. All mice were sacrificed after 18 days and the tumors were carefully dissected and snap frozen for RNA and protein expression analysis.

### Statistical analysis

The data are expressed as the mean ± Standard error of the mean (SEM) from at least three independent experiments. Comparisons between two groups were performed using Student's *t*-test, unless otherwise indicated. Statistical analyses were performed with the SPSS 13.0 statistical software package (SPSS Inc. Chicago, IL, USA). All statistical tests were two-sided, and *P* < 0.05 was considered to be statistically significant.

## SUPPLEMENTARY FIGURES AND TABLES


