# Supplementary material for: Gold nano-particles (AuNPs) carrying anti-EBV-miR-BART7-3p inhibit growth of EBV-positive nasopharyngeal carcinoma
Source: Oncotarget. 2015 Feb 19;6(10):7838–50. doi: 10.18632/oncotarget.3046 (PMC4480720; doi:10.18632/oncotarget.3046)
Supplement: Supplementary file 1 [file oncotarget-06-7838-s001.pdf]

SUPPLEMENTARY FIGURES AND TABLES

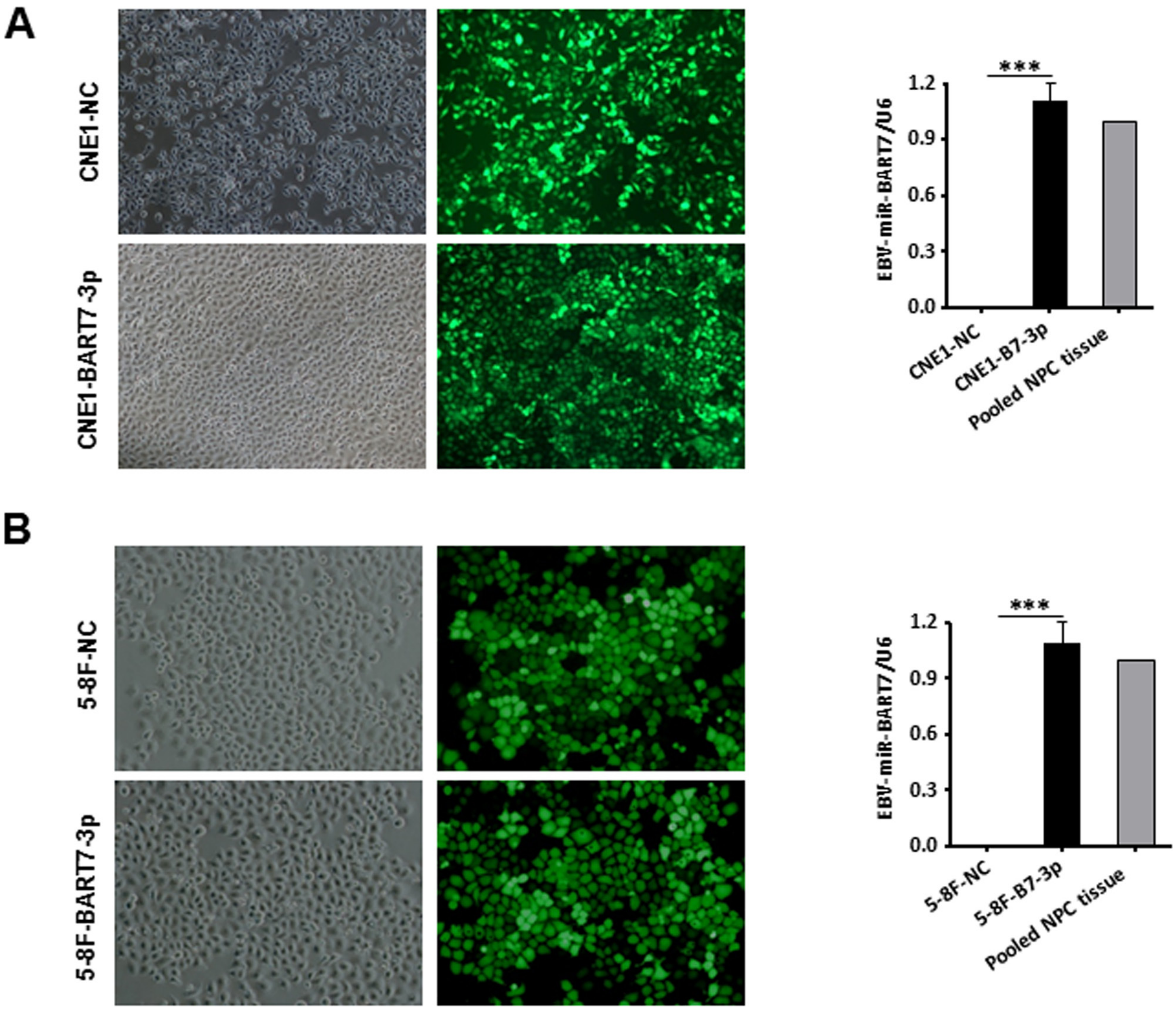

Supplementary Figure S1: NPC cell lines (GFP+) stably expressing EBV-miR-BART7–3p (5–8F-BART7–3p and CNE1-BART7–3p cells) were generated using lentivirus infection. **(A, B)** 5–8F-BART7–3p cells and CNE1-BART7–3p cells were screened respectively by GFP using FACS cytometer. qPCR validated EBV-miR-BART7–3p expression in 5–8F-BART7–3p, CNE1-BART7–3p cells compared to their corresponding control cells (5–8F-NC and CNE1-NC) and clinical NPC tissue sample (5 samples were pooled). Data were plotted as mean values ± SEM (***$P < 0.001$).

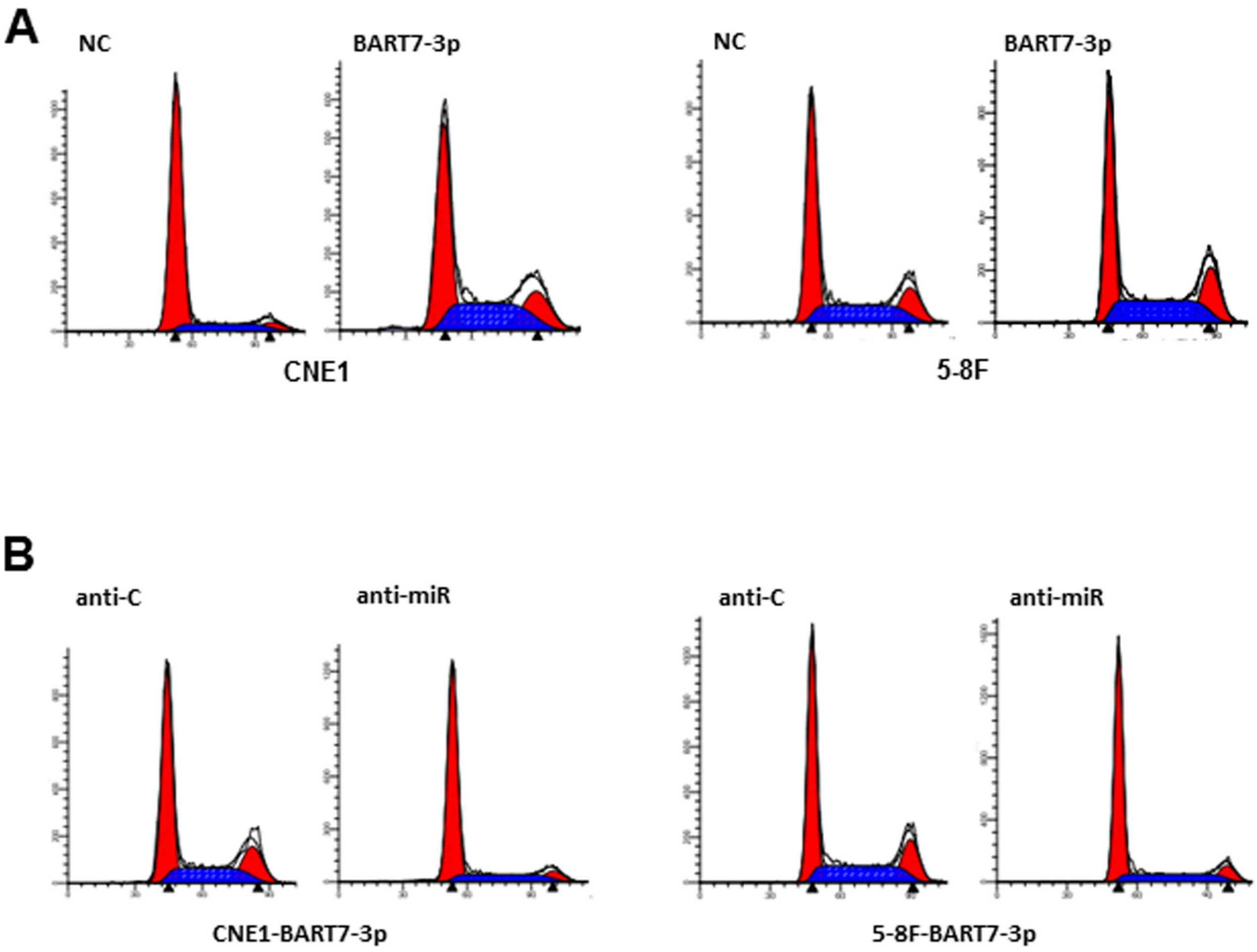

Supplementary Figure S2: Cell cycle distribution was tested by flow cytometry. **(A)** Cell cycle distribution was tested by flow cytometry in CNE1-BART7–3p and 5–8F-BART7–3p cells. **(B)** Cell cycle distribution was tested by flow cytometry in two stable NPC cells after the treatment of anti-miR.

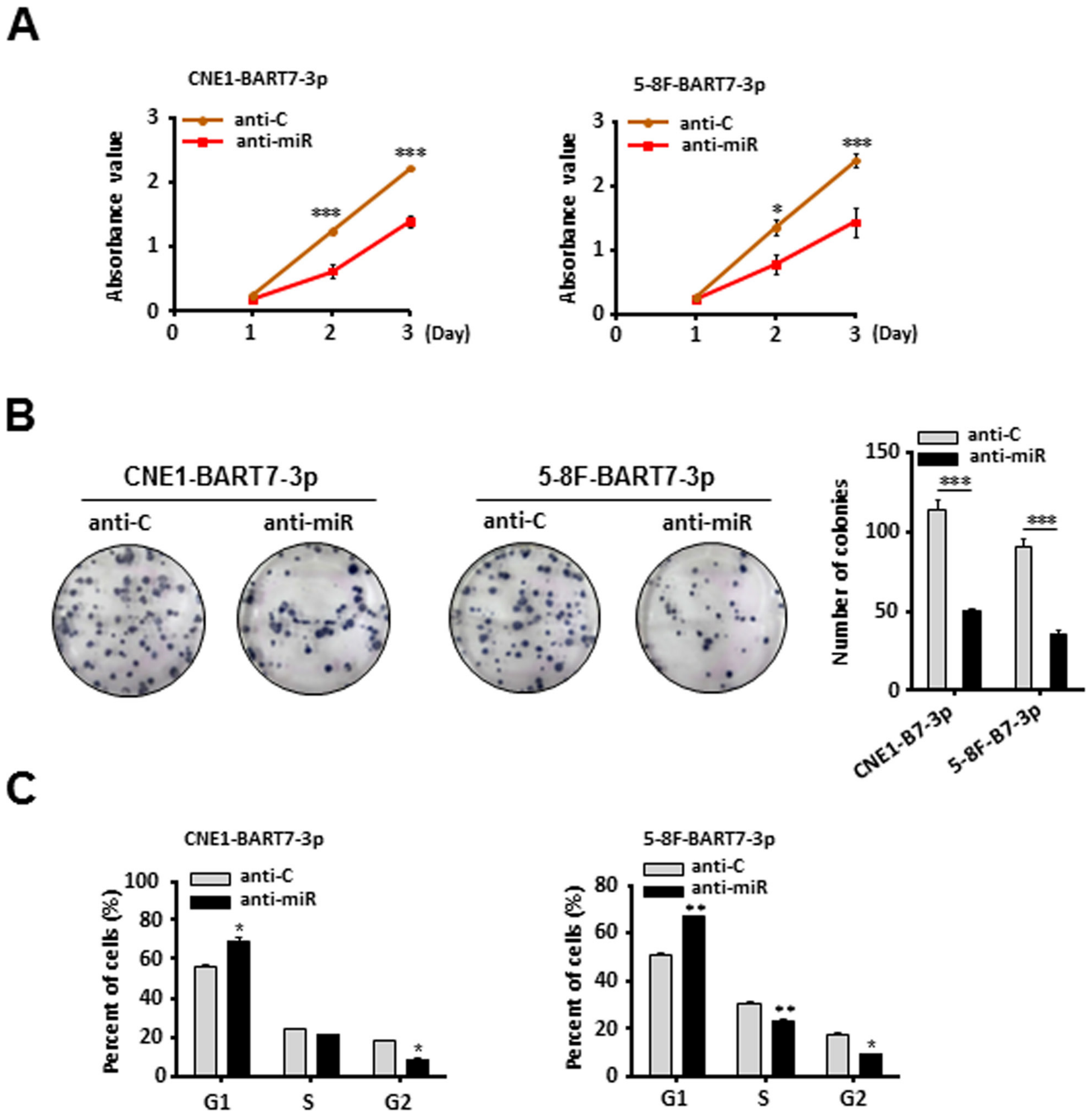

Supplementary Figure S3: The growth and proliferation was significantly inhibited in CNE1-BART7–3p and 5–8F-BART7–3p cells following the treatment of anti-miR. **(A)** Assessment of proliferation in CNE1-BART7–3p and 5–8F-BART7–3p cells after the treatment of anti-miR by MTT assay. Data are plotted as mean values ± SEM (*$P < 0.05$, ***$P < 0.001$). **(B)**,**(C)** The colony formation ability and cell cycle distribution in CNE1-BART7–3p and 5–8F-BART7–3p cells after the treatment of anti-miR. Data are plotted as mean values ± SEM (*$P < 0.05$; **$P < 0.01$; ***$P < 0.001$).

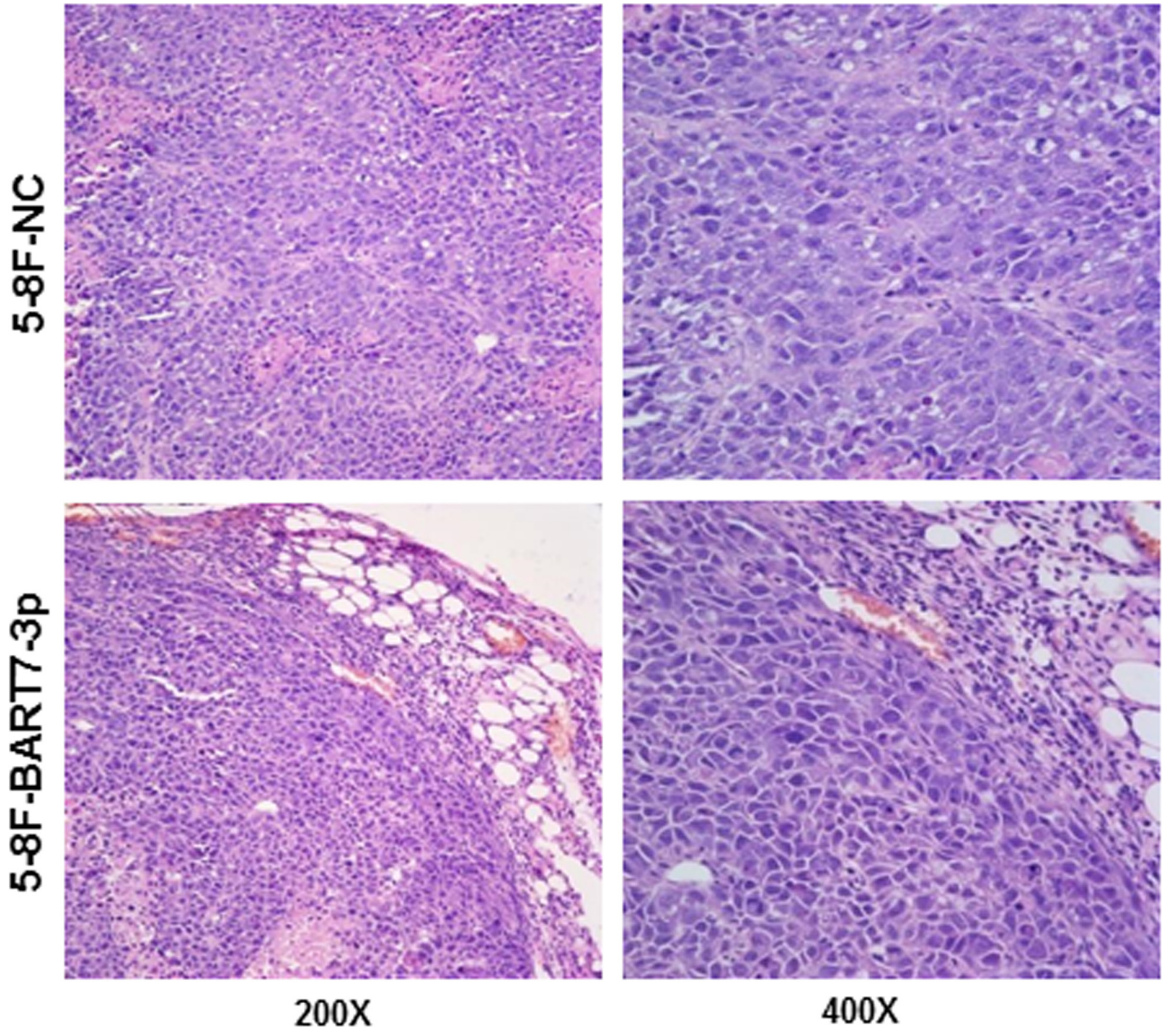

Supplementary Figure S4: HE staining confirmed xenograft tumor. Original magnification, × 200, × 400.

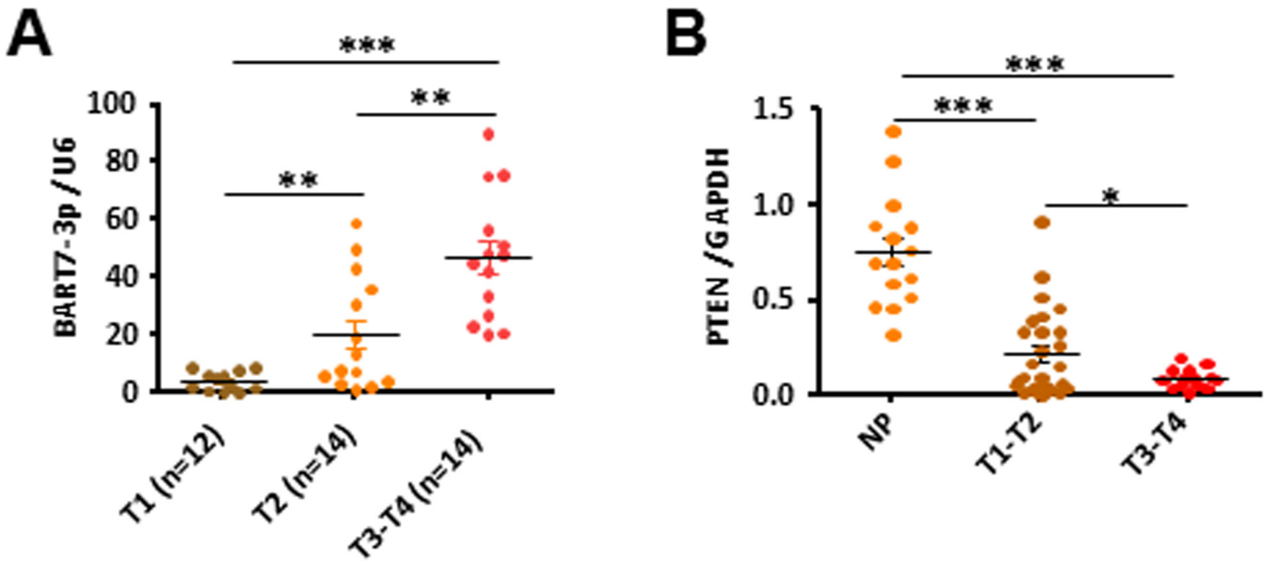

Supplementary Figure S5: The expression of EBV-miR-BART7–3p and PTEN in clinical NPC samples. (A) The expression level of EBV-miR-BART7–3p normalized to U6 snRNA was tested by qPCR in clinical NPC tissue samples with clinical T stage. **(B)** PTEN expression normalized to GAPDH expression was detected by qPCR in NPC samples with T stage information and NP samples. The data were shown as the mean ± SEM (*$P < 0.05$, **$P < 0.01$, ***$P < 0.001$).

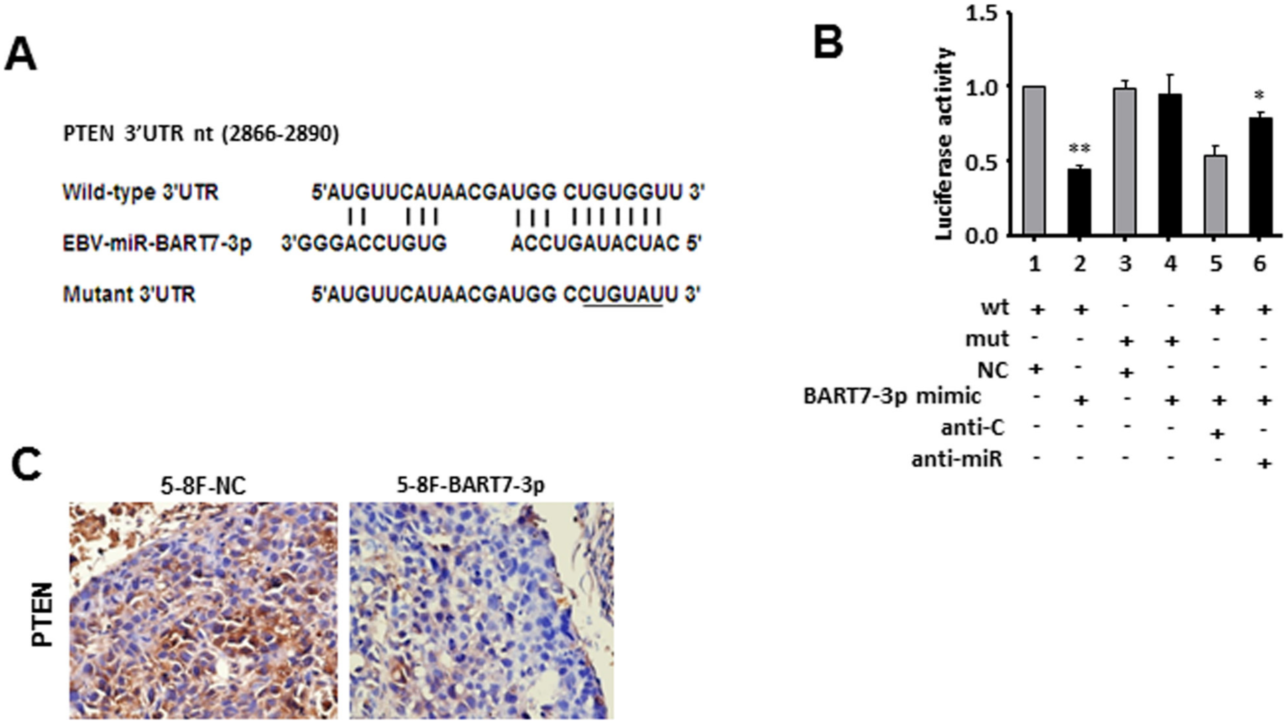

Supplementary Figure S6: PTEN was further validated to be a target of EBV-miR-BART7–3p. (A) A new putative binding site was predicted in the 3’UTR of PTEN by RNAhybrid. **(B)** EBV-miR-BART7–3p mimic or NC were cotransfected with luciferase reporters carrying the wild or the mutant binding site within PTEN 3’-UTR (wt) into 293T cells. Additionally, EBV-miR-BART7–3p mimic were cotransfected with anti-miR or anti-C and luciferase reporters carrying the wild-type binding site into 293T cells. Data were plotted as mean values ± SEM (*$P < 0.05$, **$P < 0.01$). **(C)** PTEN expression was evaluated by IHC in tumor tissues derived from NPC models compared with control models. Original magnification, × 400.

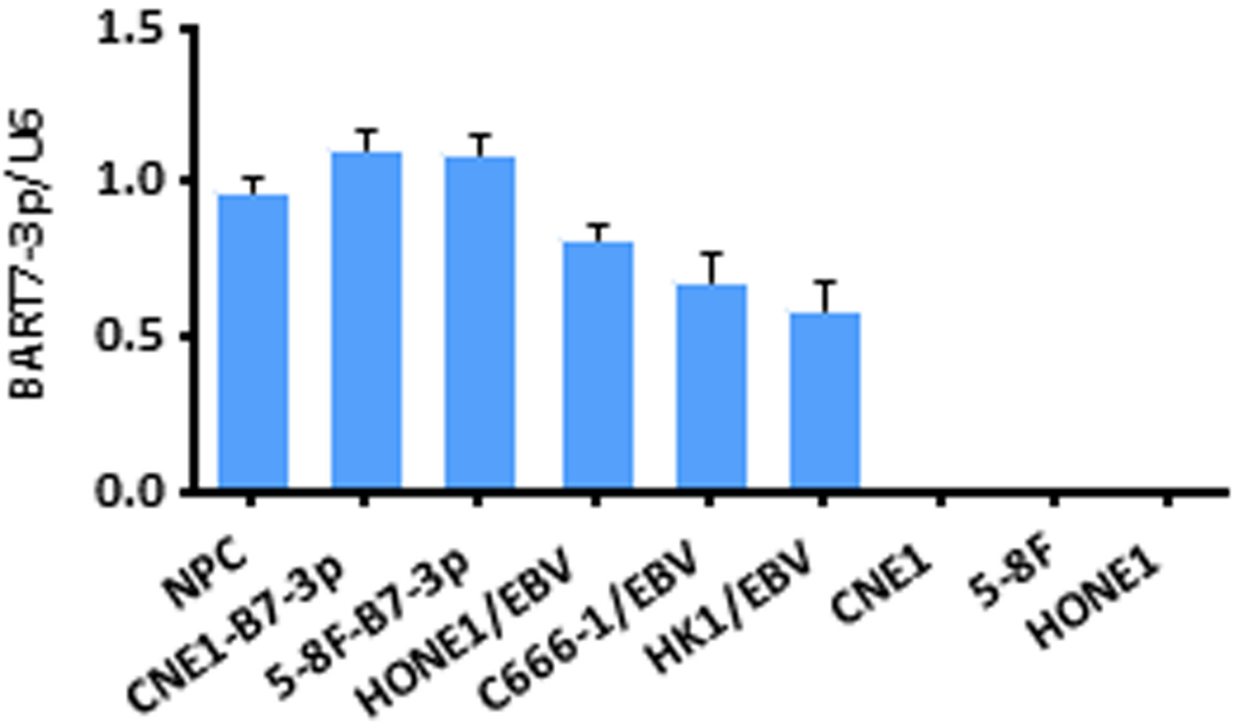

Supplementary Figure S7: The detection of EBV-miR-BART7–3p expression in NPC cell lines. EBV-miR-BART7–3p expression was examined by qPCR in three NPC EBV-positive cells, pooled NPC tissues (5 samples were pooled), CNE1-BART7–3p, 5–8F-BART7–3p cells and three EBV-negative NPC cell lines. Data were plotted as mean values ± SEM.

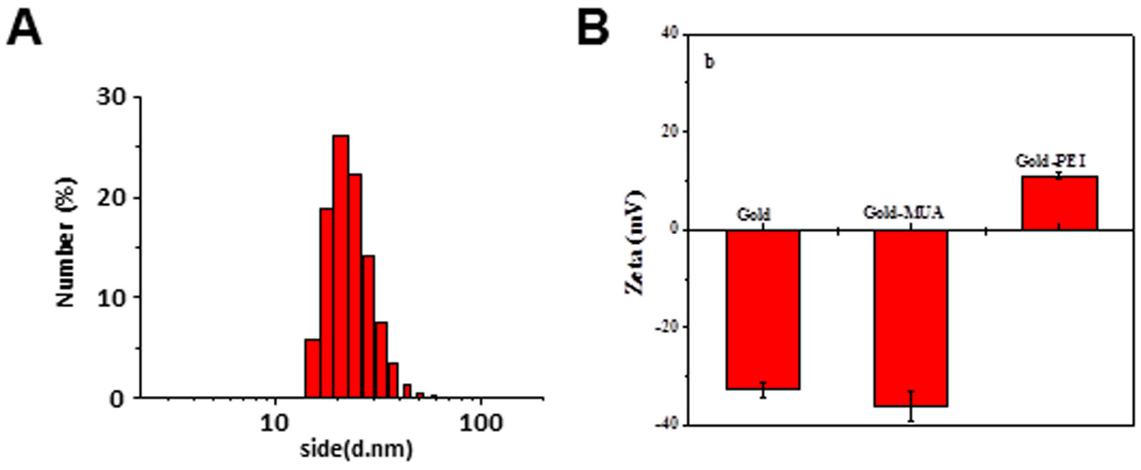

Supplementary Figure S8: The size of Gold-PEI nano-carrier. **(A)** The size of Gold-PEI nano-carrier was measured by dynamic lighting scatter (DLS). **(B)** The zeta potential of gold-PEI nano-carrier was positive charge.

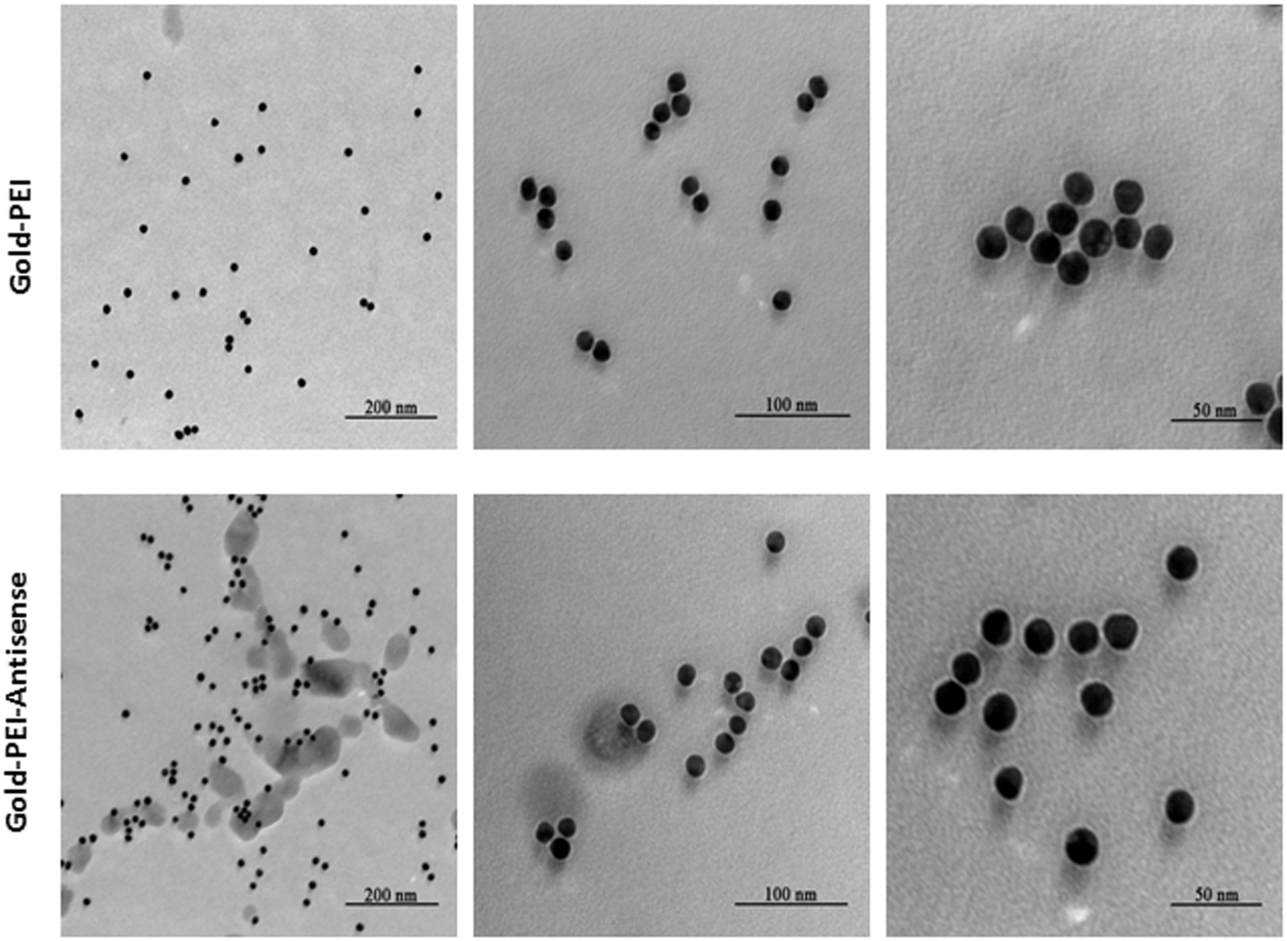

Supplementary Figure S9: Gold-PEI nano-carrier morphology. The size and shape of Gold-PEI nano-carrier were confirmed by the transmission electron microscopy (TEM).

Supplementary Table S1: The information of Clinical samples for clinical data analysis

Characteristics	Clinical samples		
	NP (*n* = 15)	NPC (*n* = 40)	*p*-value
Age, years	41.67	44.22	0.4640*
Gender, male	8	32	0.0861&
Poorly differentiated SCC		1	
Undifferentiated cancer		39	
Differentiated SCC		0	

Supplementary Table S2: Clinicopathological characteristics of patients with NPC

Characteristic	No of patients ($N = 40$)
Sex	
Male	32
Female	8
Age (years)	
≤ 45	26
> 45	14
Histological type	
Poorly differentiated SCC	1
Undifferentiated cancer	39
Differentiated SCC	0
T stage	
T1	12
T2	14
T3	6
T4	8
N stage	
N0	9
N1	9
N2	12
N3	10
M stage	
M0	38
M1	1
M2	1
M3	0
TNM stage	
I	4
II	9
III	14
IV	13

Supplementary Table S3: RNA oligoribonucleotides for EBV-miR-BART7–3p

Mimic and anti-miR	Sense strand (5′-3′)
mimic	CAUCAUAGUCCAGUGUCCAGGG
NC	UUCUCCGAACGUGUCACG
anti-miR	CCCUGGACACUGGACUAUGAUG
anti- C	CAGUACUUUUGUGUAGUACAA

Supplementary Table S4: Primers for qRT-PCR assay of cell cycle regulators

Gene	Forward primer (5′-3′)	Reverse primer (5′-3′)
CCND1	GCTCCTGGTGAACAAGCTCAA	TTGGAGAGGAAGTGTTCAATGAAA
CCND2	TCATGACTTCATTGAGCA	CACTTCCTCATCCTGCTG
CDK4	TTCGTGAGGTGGCTTTACTG	GATATGTCCTTAGGTCCTGGTCT
CCNE1	CTTCACAGGGAGACCTTTTAC	CATTCAGCCAGGACACAATAG
CCNA1	TAGACACCGGCACACTCAAG	AGGAGAGATGAATCTACCAGCAT
CDK2	TTCTGCCATTCTCATCGG	ATGGGTGTAAGTACGAACAGG
CCNB1	AACTTTCGCCTGAGCCTATTTT	TTGGTCTGACTGCTTGCTCTT
CDK1	CTAACAGCAGAGAGCGTCACC	AAAGGTTTGATAACTGTGCCCA
$p15^{INK4B}$	GTTTTGGCGACCCCTGTAGAC	GCATTCCACCAGACAAACTATCA
$p16^{INK4A}$	ACCGAATAGTTACGGTCGGAGG	CATCATCATGACCTGGTCTTCTAGG
$p21^{CIP1}$	GGCAGACCAGCATGACAGATT	GCGGATTAGGGCTTCCTCTT
$p27^{KIP1}$	AAAGCACTCAGCAGATGGGTT	GATGCACGTTCCAGAGTTTCG
pRb	GCAGTATGCTTCCACCAGGC	AAGGGCTTCGAGGAATGTGAG
GAPDH	GGTGAAGGTCGGAGTCAACGGA	GTCATGGATGACCTTGGCCAGG

Supplementary Table S5: Primers for qRT-PCR assay of miRNA and PTEN

Primers	Sense strand (5′-3′)
EBV-miR-BART7–3p	CATCATAGTCCAGTGTCCAGGG
U6	CTCGCTTCGGCAGCACATATA
PTEN Forward	TGTGGTCTGCCAGCTAAAGG
PTEN Reverse	CGGCTGAGGGAACTCAAAGT
GAPDH Forward	GGTGAAGGTCGGAGTCAACGGA
GAPDH Reverse	GTCATGGATGACCTTGGCCAGG

Supplementary Table S6: The information of antibodies

Antibody	Cat. No.	Company	Molecular weight	Dilution(WB/IHC)
PTEN	BS1305	Bioworld	54kDa	1:800/1:200
Akt	BS2987	Bioworld	55kDa	1:1000
Akt(p-Ser473)	AM1006	ABZOOM	60kDa	1:1000
c-Myc	1472–1	Epitomics	57kDa	1:1000
c-Jun	1696–1	Epitomics	43kDa	1:1000
Cyclin D1	10438–1-AP	proteintech	34kDa	1:800
Cyclin E1	1655–1	Epitomics	47kDa	1:800
p21	3733–1	Epitomics	21kDa	1:1000
Ki67	ab16667	Abcam		1:400
PCNA	A0264			1:100
Beta-actin	Jan-79	Epitomics	43kDa	1:1000
